# Metabolic flux analysis of heterotrophic growth in *Chlamydomonas reinhardtii*

**DOI:** 10.1371/journal.pone.0177292

**Published:** 2017-05-24

**Authors:** Nanette R. Boyle, Neelanjan Sengupta, John A. Morgan

**Affiliations:** School of Chemical Engineering, Purdue University, West Lafayette, Indiana, United States of America; Universidade Federal de Vicosa, BRAZIL

## Abstract

Despite the wealth of knowledge available for *C*. *reinhardtii*, the central metabolic fluxes of growth on acetate have not yet been determined. In this study, ^13^C-metabolic flux analysis (^13^C-MFA) was used to determine and quantify the metabolic pathways of primary metabolism in *C*. *reinhardtii* cells grown under heterotrophic conditions with acetate as the sole carbon source. Isotopic labeling patterns of compartment specific biomass derived metabolites were used to calculate the fluxes. It was found that acetate is ligated with coenzyme A in the three subcellular compartments (cytosol, mitochondria and plastid) included in the model. Two citrate synthases were found to potentially be involved in acetyl-coA metabolism; one localized in the mitochondria and the other acting outside the mitochondria. Labeling patterns demonstrate that Acetyl-coA synthesized in the plastid is directly incorporated in synthesis of fatty acids. Despite having a complete TCA cycle in the mitochondria, it was also found that a majority of the malate flux is shuttled to the cytosol and plastid where it is converted to oxaloacetate providing reducing equivalents to these compartments. When compared to predictions by flux balance analysis, fluxes measured with ^13^C-MFA were found to be suboptimal with respect to biomass yield; *C*. *reinhardtii* sacrifices biomass yield to produce ATP and reducing equivalents.

## Introduction

Rising levels of carbon dioxide in the atmosphere have increased the need to develop more sustainable sources of feedstocks traditionally supplied by petroleum. Although there are many options for renewable energy, certain sectors (such as transportation) still require liquid fuels. One of the more promising sources of liquid transportation fuel is algae derived biodiesel [1, 2], which has higher energy density than ethanol and does not compete with food or feed supplies for resources, especially when brackish water can be used for cultivation. Despite increased interest in the development of algal-derived fuels, there is a general lack of knowledge of algal biology which has delayed attempts to engineer these organisms for increased production of fuels. One of the most prominent model systems, Chlamydomonas reinhardtii, has long been used for photosynthesis and chloroplast biogenesis research [3, 4] and was one of the first algal genomes sequenced [5]. Until recently, it was also the only green algae species in which sophisticated metabolic engineering could be attempted due to a general lack of tools for other species [6]. Despite the wealth of knowledge, much remains in order to understand basal in vivo carbon fluxes, which is a first step in rational metabolic engineering.

The ability to quantify intracellular metabolic fluxes provides researchers a systems level view of genetic and environmental effects on metabolism. Flux balance analysis (FBA) is a mathematical modeling technique for predicting intracellular carbon fluxes using stoichiometric balances, uptake rates of nutrients as constraints and optimization principles such as maximizing the cellular growth rate to estimate intracellular fluxes. FBA has been applied to a variety of model photosynthetic organisms, including (but not limited to) *Synechocystis* sp. PCC 6803 [7–9], *Synechococcus* sp. PCC 7002 [10, 11], *Cyanothece* sp. ATCC 51142 [12], *C*. *reinhardtii* [13–17], *Chlorella* sp. FC2 IITG [18], *Chlorella protothecoides* [19], and *Arabidopsis thaliana* [20] to estimate fluxes and yields. However, despite its widespread use to predict fluxes in large-scale networks, studies have shown that FBA is not always accurate in predicting *in vivo* fluxes [21, 22]. A variety of modifications have been added to the classical formulation of FBA to account for non-optimal growth [22–25], including thermodynamic and regulatory constraints. Despite the development of these more complex FBA models, isotope assisted metabolic flux analysis (MFA) is the standard technique used to quantify intracellular fluxes because the additional data (in the form of isotopic labelling of metabolites and flux split ratios) allows measurement of fluxes in the cell instead of assuming optimization of an objective function [26, 27]. ^13^C metabolic flux analysis (^13^C-MFA) is a powerful technique that utilizes *in vivo* isotopic labeling patterns of metabolites and mathematical modeling to quantify fluxes. MFA has been applied to a variety of organisms, including crops such as rice [28] and soybean [29–31] and has been also used to distinguish various network topologies and quantify the flux through parallel pathways in plants, such as the presence of glycolytic and pentose phosphate (PPP) pathways in both the cytosol and plastid in Arabidopsis [32], *Brassica napus* [33] and Maize [34–36]. More recently, ^13^C-MFA has been used to investigate heterotrophic fluxes of the green alga *Chlorella protothecoides* in nitrogen replete and limited conditions [19, 37].

The main goal of this study is to investigate heterotrophic growth of *C*. *reinhardtii* with ^13^C labeled acetate and employ ^13^C–MFA to answer key qualitative and quantitative questions regarding acetate assimilation and utilization. As an acetate flagellate, the only carbon source which can be metabolized by *C*. *reinhardtii* in the absence of light is acetate and other two carbon molecules; therefore, quantifying metabolic fluxes for growth on acetate is integral to understanding heterotrophic carbon fluxes in *C*. *reinhardtii*. The exact mechanism of acetate assimilation in *C*. *reinhardtii* is yet unknown, but is generally thought to be transported into the mitochondrion, and assimilated via the glyoxylate shunt [4]. According to version 5.5 of the Chlamydomonas genome draft at JGI [5], Chlamydomonas has three acetyl-CoA synthetase genes (*ACS1*, *ACS2*, *ACS3*) and two acetate kinase genes (*ACK1*, *ACK2*) which encode the enzymes responsible for initiating the conversion of acetate into Acetyl-CoA. The distributed sub-cellular localization of these enzymes and experimental evidence indicates that acetate can be transported into any of the three major compartments in the cell for assimilation (i.e. cytosol, mitochondrion and the plastid). *C*. *reinhardtii* also possesses the genes for the glyoxylate shunt, isocitrate lyase (*ICL*) and malate synthase (*MAS1*), which are critical for 2 carbon substrate utilization and are both targeted to mitochondria. Blaby et al. report the activity of ICL and MAS1 in Chlamydomonas grown in TAP medium, with dramatic increases when cells are starved for nitrogen, a condition that results in increased triacylglycerol accumulation [38]. One of the first steps of AcCoA assimilation is the reaction between oxaloacetate and AcCoA to form citrate by citrate synthase; *C*. *reinhardtii* has two citrate synthase genes, *CIS1* and *CIS2*. *CIS1* is predicted to be mitochondrial [39], while *CIS2* is predicted to be non-mitochondrial due to the presence of a different target peptide sequence [40]. We compared fluxes calculated by ^13^C-MFA to those predicted by the previously reported FBA model developed by our lab [13] to gain insight into cellular physiology and how fluxes deviate from optimal fluxes distributions.

## Materials and methods

### Culture conditions

*Chlamydomonas reinhardtii* (CC-400 *cw15* mt^+^) was obtained from the Chlamydomonas Genetics Center (Duke University, Durham, NC). Cells were cultivated in a 1.5 L Bioflo 3000 from New Brunswick Scientific (Edison, NJ) with a working volume of 1.3 liters in Tris-acetate-phosphate (TAP) medium [4]. The composition of TAP medium is given in Table 1. To prevent light from penetrating into the reactor, the surface was covered with aluminum foil. A mixture of labeled and unlabeled acetic acid (Sigma St. Louis, MO) was used in the medium in the following ratio (w/w) 0.4 unlabeled acetic acid: 0.3 [1-^13^C] acetic acid: 0.3 [^13^C_2_] labeled acetic acid. Cells were cultivated at 25°C, 250 rpm and sparged with air at a rate of 1 vvm. Cells were grown in pH-stat mode at pH 7.5, which was controlled using 20% NH_4_OH and 20% acetic acid in the same isotopic ratio as the original media. Therefore, the concentration of acetate in the reactor was maintained at a constant concentration and isotopic ^13^C ratio throughout the growth period. Acetate uptake was monitored by tracking acetate addition to the reactor. Cells were sampled in mid-exponential phase growth, as indicated in S1 Fig. Growth and yield data are provided in S2 Table. The acetate uptake rate was 0.26 mmol/hr.

**Table 1:** Brief overview of the macromolecule of interest and how each provides distinct information of compartment specific labelling.

Cellular Component	Molecules	Compartment(s)
Starch	hexoses	plastid
Cell Wall	xylose, arabinose, galactose and mannose	cytosol
Lipids	palmitic and oleic acids	plastid (acetyl-CoA)
Protein	proline, threonine, leucine, isoleucine, phenylalanine	plastid
	glutamate	mitochondria
	alanine	cytosol/plastid
	aspartate	mitochondria/plastid

### Sampling and extraction

Cells were sampled and processed in a modified procedure from that reported in Lee and Fiehn [41]. Cells were harvested by injecting 15 ml of cell suspension from the reactor into 30 ml of -70°C methanol kept in a dry ice/ethanol bath to maintain a temperature below -20°C. Samples were then centrifuged at 10,000 rpm for 10 minutes in a prechilled rotor at -20°C. The supernatant was removed and the cell pellet was flash frozen in liquid nitrogen. The frozen cell pellet was then transferred to a ball mill MM 400 (Retsch GmbH & Co., Germany) with a single stainless steel ball (i.d. = 5 mm). One ml of cold (-70°C) methanol was added to the grinder chamber which was then immersed in liquid nitrogen for approximately 1 minute. The grinding chamber was then placed in the ball mill and the sample was processed for 3 minutes at 15 Hz. After removing the liquid in the chamber, an additional 1 ml of cold methanol was used to rinse the chamber. The extracts and rinsing fluid were combined and then centrifuged at 10,000 rpm for 10 minutes at -20°C. The pellet was resuspended in 1 ml cold methanol, spun down and the supernatant combined with the extract from the previous step. The pellet was saved for further processing. The extract was dried in a centrivap (Labconco, Kansas City, MO) and kept at -80°C until analysis by GC/MS (to measure organic acids).

### Starch and cell wall hydrolysate

Analysis of labeling in starch and cell wall hydrolysates was analyzed as described by Allen et al. [31]. The pellet resulting from the methanol extraction was resuspended in 0.5 ml of 0.1 M acetate buffer, pH = 4.8. The solution was autoclaved for 1 hour, and allowed to cool to room temperature. A 10 mg solution of amyloglucosidase and amylase in a 2:3 ratio (Sigma, St. Louis) was added and incubated at 55°C for 1 hour. One ml of ethanol was added and the solution heated to 95°C for 15 minutes to denature the enzymes. The solution was then centrifuged at 4000 rpm for 5 minutes and the supernatant fraction (starch) was saved. A second extraction was performed by resuspending the pellet in 2 ml of 8% ethanol and pelleted again. The supernatant was combined with that saved in the first step and dried in a centrivap. The pellet was then resuspended in 500 μl 2 M trifluoroacetic acid and incubated at 120°C for 2 hours. The solution was then centrifuged at 10,000 rpm for 5 minutes and the supernatant removed. 500 μl 0.5 M NH_4_OH was then added to the supernatant and dried in a centrivap and kept at -80°C until analysis by GC/MS (to measure sugars present in starch and the cell wall).

### Hydrolysis of proteins

Cell pellets resulting from the methanol extraction step was resuspended in 1 ml 6 M HCl and heated at 120°C for 24 hours under vacuum to hydrolyze the proteins to amino acids. The solution was then dried in a centrivap and kept at -80°C until analysis by GC/MS (to measure amino acid composition and concentrations).

### Derivatization

Samples for organic acids, amino acids and sugars were derivatized for GC/MS analysis. First, dried samples were resuspended in 100 μl methoxyamine HCl in pyridine (20 mg/ml) and placed in a heating block at 40°C for 90 minutes. Immediately following this, 100 μl of either trimethylsilane (TMS) or t-butyldimethylsilyl (TBDMS) was added; samples were vortexed and then, placed in a heating block at 40°C for 30 minutes. For the analysis of sugars and organic acids, the derivatization agent used was TMS; for the analysis of amino acids, TBDMS was used. The TMS derivatized sugars produced characteristic fragment ions that contain information on positional labeling of the carbon atoms [42].

### Fatty acids

Dried methanol extracts were resuspended in 600 μl hexane to which 500 μl butylamide was added. The reaction was allowed to proceed for one hour at 40°C, after which the reaction was quenched by drop-wise addition of 4M HCl. The samples were then vortexed, the organic layer was removed and the aqueous layer was extracted again with 500 μl hexane. After vortexing, the organic layer was removed and combined with the first extract and dried. The dried butylamide derivitaives were then resuspended in 300 μl hexane and analyzed with GC/MS.

### Analytical methods

GC-MS analysis was carried out on an Agilent 7890A gas chromatograph coupled to an Agilent 5975C inert MSD quadrupole mass spectrometer. The GC was equipped with a HP-5MS column (0.25mm x 30m x 0.25μm, Agilent, Santa Clara, CA) with helium as the carrier gas. Interface and ion source temperatures were both 230°C. Amino acids were analyzed with a split-less 1μl injection. Oven temperature was initially held at 135°C for 3 minutes followed by a ramp of 2.5°C/minute up to a temperature of 280°C for 2 minutes. Sugars in cell wall hydrolysate and starch samples were analyzed with a 1μl injection 10:1 split ratio. The oven temperature was initially held at 150°C for 1 minute followed by a ramp of 5°C/minute up to a temperature of 175°C followed by a ramp of 3°C /minute up to a temperature of 240°C where it was held for 2 minutes followed by a final ramp of 10°C /minute up to 250°C. Fatty acids were analyzed with a split-less 1μl injection. Oven temperature was initially held at 80°C for 3 minutes followed by a 30°C/min ramp up to 200°C where it was held for 2 minutes followed by another ramp of 6°C/min up to 305°C. Labeling patterns of metabolites, amino acids, fatty acids and sugars used in the model are given in the supplementary material, S3–S7 Tables.

### Mathematical modeling

#### Flux balance analysis

The previously reported stoichiometric model for *C. reinhardtii* [13] was used to predict intracellular fluxes for two objective functions. The yield measured during pH-stat growth for this experiment, 16.6 g DW per mole acetate, differed from that previously reported from a batch growth study and thus the maintenance cost was adjusted to fit the model to the experimental yield. The maintenance cost, in terms of ATP, increased from 29.9 mmol ATP/g biomass to 140 mmol ATP/g biomass. Maximum biomass for heterotrophic growth was simulated as previously described [13]. The objective function of maximum ATP production was performed by maximizing the sum of fluxes through ATP hydrolysis reactions (ν167, ν168, ν169). Acetate uptake was constrained to the same value used for 13C-MFA calculations, 0.26 mmol/hr. Starch and lipids were allowed to accumulate in both simulations. The coefficients for precursors required to produce 1 kg biomass are given in S8 Table.

#### Flux evaluation with isotopomer balancing

**Fig 1:**
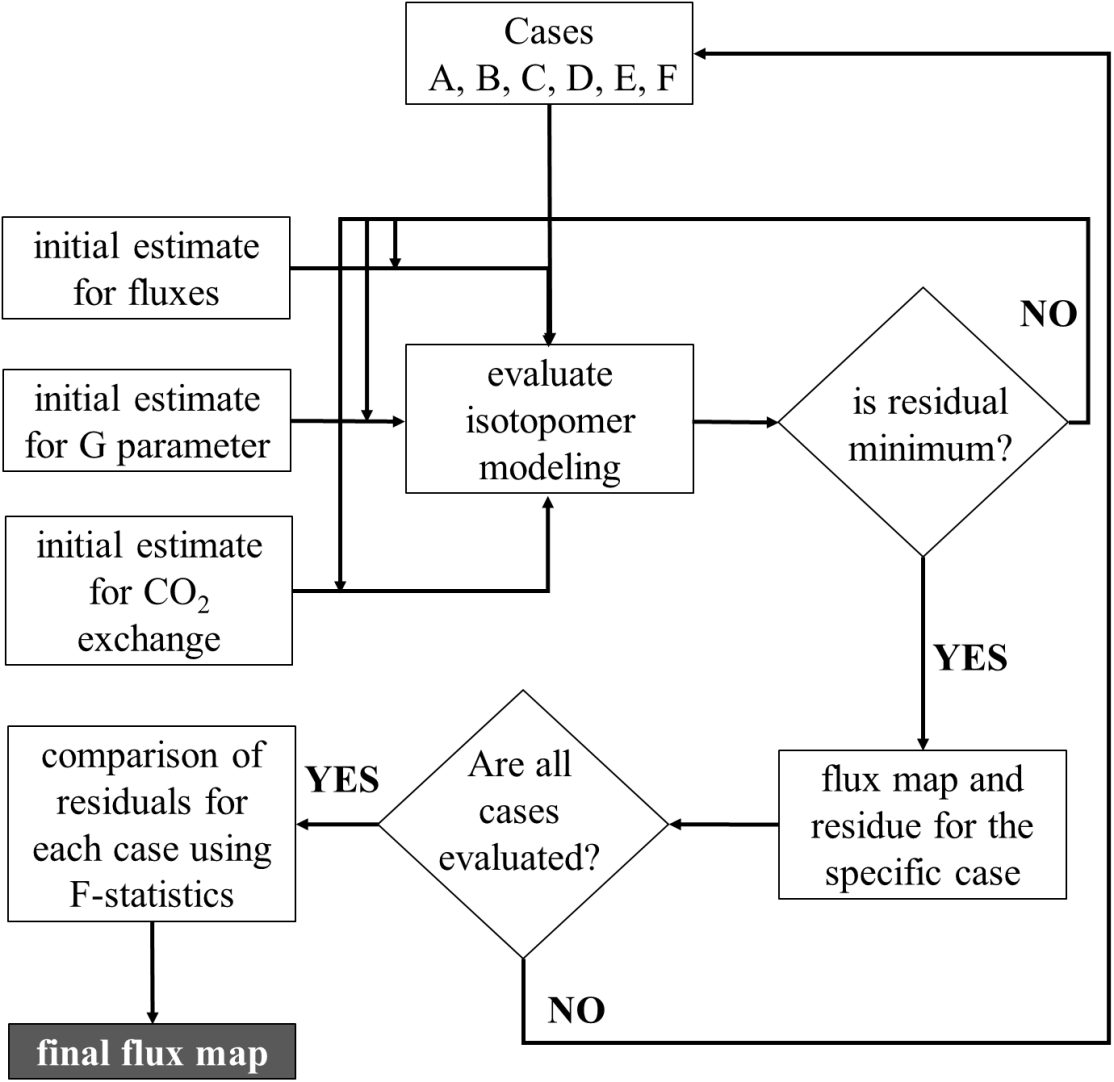
Schematic of the overall modeling strategy. All the cases were evaluated independently and were evaluated by including dilution due to existing unlabeled biomass and unlabeled CO_2_ from air. The case that gave the best statistical fit was selected as the final flux map for heterotrophic growth on acetate.

In general, the process is to identify the set of reactions and corresponding fluxes that are best supported by the 13C labeling data. The evaluation of alternative network topologies was performed first followed by parameter optimization (see Fig 1). Flux evaluation was performed using isotopomer modeling similar to previously reported models [26, 27, 43, 44]. The set of isotopomer balances were solved to obtain isotopomer distribution vectors (IDV). The isotopomer modeling strategy is given in supplementary S3 Fig. The total size of the isotopomer balance was 854, and the model consisted of 44 independent parameters, of which 10 were independent fluxes, the remaining were exchange coefficients and G parameter (fractional biomass dilution parameter). There were 112 mass distribution vectors (MDVs) from MS measurements utilized in the fitting procedure. The concept of G parameter was first introduced by Antoniewicz et al. [45] to account for the dilution of the observed biomass labeling patterns due to presence of the original unlabeled biomass in batch culture. Thus G represents the fractional contribution of the actual labeled biomass towards the observed labeling patterns and 1-G is the fractional contribution of the unlabeled biomass (from inoculation) towards the observed labeling patterns. The G parameters were solved for along with the fluxes in the optimization framework described in Fig 1. Further it should be noted that for the comparison for network topologies a single G parameter was used for the entire biomass. However for the final reported flux map, different G parameters were assigned to different biomass components (lipids, cell wall, plastidic sugars and proteins). Multiple MDVs belonging to the same metabolite will have the same G parameter associated with it (as shown below). Where MDVexp_m is the experimentally observed MDV for a metabolite, MDVcalculated is the calculated MDV, derived from solving the isotopomer balances, and MDVnatural is the natural abundance.

$$MDV_{\text{exp}\_m}=MDV_{\text{calculated}}*G_{m}+MDV_{\text{natural}}*\left(1-G_{m}\right)$$ (1)

The IDVs were used to calculate the MDVs, and the calculated MDVs were used for least square optimization. The objective function was minimization of the sum of the square of the difference between the experimentally observed and simulated MDVs (see Eq 1).

$$\text{Obj}=\sum_{i=0}^{n}{\left(MDV_{cal}-MDV_{exp}\right)}_{i}^{2}$$ (2)

Where N is total number of species, MDV_cal_ was the calculated MDV and MDV_exp_ was the experimental value of a particular species. The optimization was performed in MATLAB R2009b (MathWorks, Natick, MA, USA) using fmincon that implements optimization for constrained non-linear functions. Further, since the results from non-linear optimization are dependent on initial flux estimates, different estimates were tested for obtaining the best solution. The flux model variance and 95% confidence intervals were determined with the method of Antoniewicz et al. [46]. Since we did not have replicates, the chi-square statistic could not be used for estimation of the confidence interval, instead F-statistic was used for determining the confidence intervals as described in Antoniewicz et al., 2006.

Since this was a non-weighted least squares optimization, the goodness of fit was evaluated by plot of the residual vs. MDV (see supplementary material S2 Fig). It was found that the residual vs. MDV plot was randomly distributed. The norm plot of the residues could be approximated as a normal distribution with μ = -.0774 and σ = 1.85, indicated that the residuals were normally distributed (with some deviation, as it is non-weighted). The average mean difference for the fitted MDVs observed was 1.3%. The high average mean difference could be attributed to biological experimental variation as increasing number of independent variables or flux network topology did not change the average difference (data not shown).

### Compartment specific labeling information

We obtained information on compartment specific labeling patterns based on methods described by Allen et al. [31]. The table below gives a brief overview of the source, molecule of interest and what compartment. Some metabolites can have labelling from more than one compartment, and thus an additional parameter which describes the fractional contribution from each of these compartments was included, and determined by optimization along with flux analysis. Further, for each amino acid, multiple fragments were included as described in Antoniewicz et al. [45] (see supplementary material).

### Comparison of different network topologies using F statistics

The comparison of models was done in terms of F statistics [46]. To evaluate whether inclusion or exclusion of certain model parameter has a statistically significant effect, the following equation is used for evaluating the F value.

$$F=\left[\frac{\left(\frac{R_{1}-R_{2}}{n_{2}-n_{1}}\right)}{\left(\frac{R_{2}}{p-n_{2}}\right)}\right]$$ (3)

Here, R_1_ is the residual (sum of square difference between experimental and simulated values) for the original model and R_2_ is the residual for the new model to be tested, (e.g. inclusion of G parameter, or inclusion/exclusion of reaction). The degrees of freedom for the original model and the new model are represented by n_1_ and n_2_ respectively. The number of experimental observations, which are the MDVs in our case, is represented by p. It should be noted that the F statistic value accounts for increase in degrees of freedom, and thus precludes improvement in fits as a result of overfitting. Once the F value is calculated, it is compared against critical F value, at a specified confidence level (90–99%), for testing the null hypothesis. If the calculated F value is greater than the critical value at a given confidence, it implies that the model is statistically different and fits the data better at the desired confidence level.

### Effect of dilution due to CO_2_ on labeling patterns

In this study, dissolved CO_2_ or HCO_3_^-^ are lumped and modeled as CO_2_. Intracellular CO_2_ produced by metabolic reactions was considered to be freely transported across compartments, and thus assumed to be at isotopic equilibrium inside the cell. The exchange of extracellular and intracellular CO_2_ from air was also included in the model. The CO_2_ transport was modeled as a reversible reaction and was characterized using an exchange coefficient; which is a parameter that gives a degree of reversibility for the transport. We used the method of Weichert and de Graaf for exchange coefficients [47].

## Results

### Network construction for heterotrophic growth

**Fig 2:**
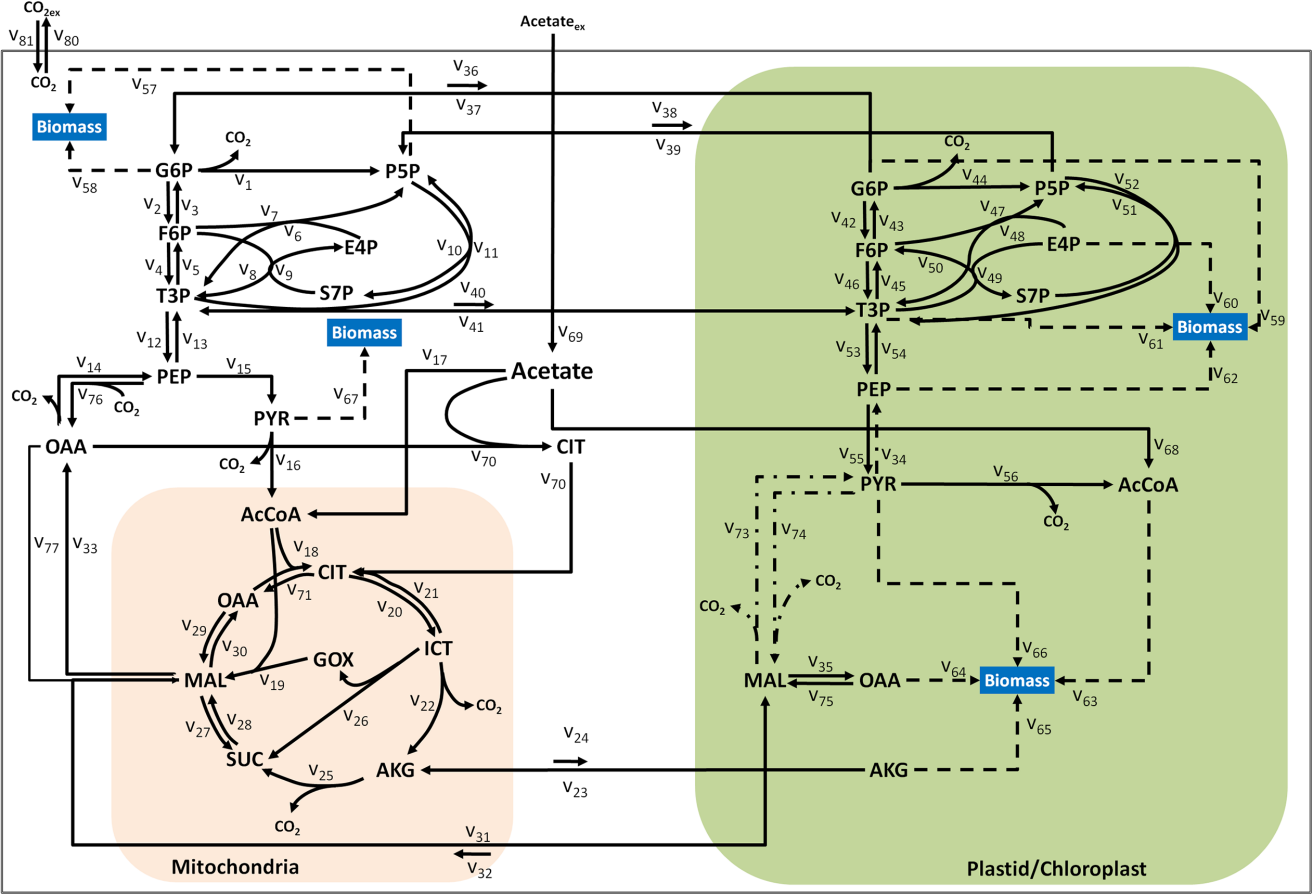
Simplified metabolic network for MFA of *Chlamydomonas reinhardtii*. The network consists of plastidic and cytosolic glycolysis and pentose phosphate pathway. The acetate imported (v_69_) can be metabolized through acetyl CoA synthetase (v_17_), cytosolic citrate synthase (v_70_) and plastidic acetyl CoA synthetase/acetate kinase (v_68_). Possible pathway for plastidic gluconeogenesis (-.-). Biomass fluxes are represented by (—). **Metabolite abbreviations**: G6P: glucose-6-phosphate, F6P: fructose-6-phosphate, T3P: Dihydroxyacetone phosphate and 3-phosphoglycerate, P5P: Pentose Phosphates: Ribulose-5-phosphate, Xylulose-5-phosphate, AcCoA: Acetyl-coA, PEP: phospho*enol* pyruvate, Pyr: pyruvate, CIT: Citrate, ICT: isocitrate, AKG: alpha-ketoglutarate, SUC: succinate, MAL: Malate, OAA: oxaloacetate, GOX: glyoxylate, E4P: erythrose-4-phosphate, S7P:sedoheptulose-7-phosphate.

Previously reported network reconstructions of *C*. *reinhardtii* [13, 14] [15–17], included hundreds of metabolic and transport reactions. The goal of this work was to identify fluxes in primary metabolism, therefore, we simplified the metabolic network reconstruction first reported by Boyle and Morgan [13] to be used as the basis for ^13^C-metabolic flux analysis (^13^C-MFA) (see Fig 2). Reactions were lumped based on published models for plant systems [29, 33] and on the analysis of compartment specific labeling information, which was used to distinguish compartment-specific reactions [31]. The simplified ^13^C-MFA model included three compartments: cytosol, plastid and mitochondria. There are two main enzymatic routes for acetate assimilation in Chlamydomonas; the first is through acetyl-CoA synthetase and the second is the coordinated effort of acetate kinase and phosphate acyltransferase; however, it is not possible to distinguish these from one another from isotopic labeling; therefore these steps were lumped together in the simplified model. The Chlamydomonas genome encodes two citrate synthases, CIS1 and CIS2. CIS1 is predicted to be mitochondrial [39], and CIS2, which possesses a different targeting sequence is predicted to be non-mitochondrial and perhaps glyoxysomal based on sequence homology to plants [40]. However, the presence of glyoxysomes in Chlamydomonas is still uncertain [48]. Moreover, there is no evidence of a chloroplast targeting peptide in CIS2 [49] and citrate synthase activity in *C*. *reinhardtii* was reported only in cytosolic fractions [50]. Therefore, this non-mitochondrial citrate synthase was localized to the cytosol in the mathematical model.

Gluconeogenesis is necessary for growth on acetate; therefore, we also closely examined the localization of enzymes in this pathway. Two splice variants of phosphoenolpyruvate carboxykinase, PCK1a and PCK1b, have been reported in *C*. *reinhardtii*. PCK1b is predicted by TargetP to be targeted to the cytosol [49] and the signal peptide for PCK1a is not definitive and may be chloroplastic or mitochondrial according to TargetP and iPSORT [51]. Enzymes for a second gluconeogenic pathway, located in the plastid, are also encoded in the genome [40]. This pathway includes the conversion of malate to pyruvate by malic enzyme (MME4) followed by the conversion of pyruvate to PEP catalyzed by pyruvate phosphate dikinase. Two isoforms of pyruvate phosphate dikinase, PPD1 and PPD2 have been reported in *C*. *reinhardtii*, of which PPD2 is most likely chloroplastic based on homology to *Flaveria pringlei* and TargetP predictions [40, 49]. In either case, these two alternative routes in the plastid result in the same carbon atom transitions and hence are indistinguishable by ^13^C tracing and hence are represented by one reaction in the model. Due to strong evidence for the presence of these reactions in the plastid, plastidic gluconeogenesis was also included in the model. Other reactions included in the simplified network include: glycolysis and pentose phosphate pathways in both the cytoplasm and plastid, TCA cycle and glyoxylate shunt in mitochondria, transport of hexoses, pentoses, trioses and select TCA cycle intermediates across compartments [13]. CO_2_ fixation by RuBisCO was not included because the culture was grown heterotrophically (without light on acetate) and therefore RuBisCO would not be active [52].

### Metabolic flux analysis

**Fig 3:**
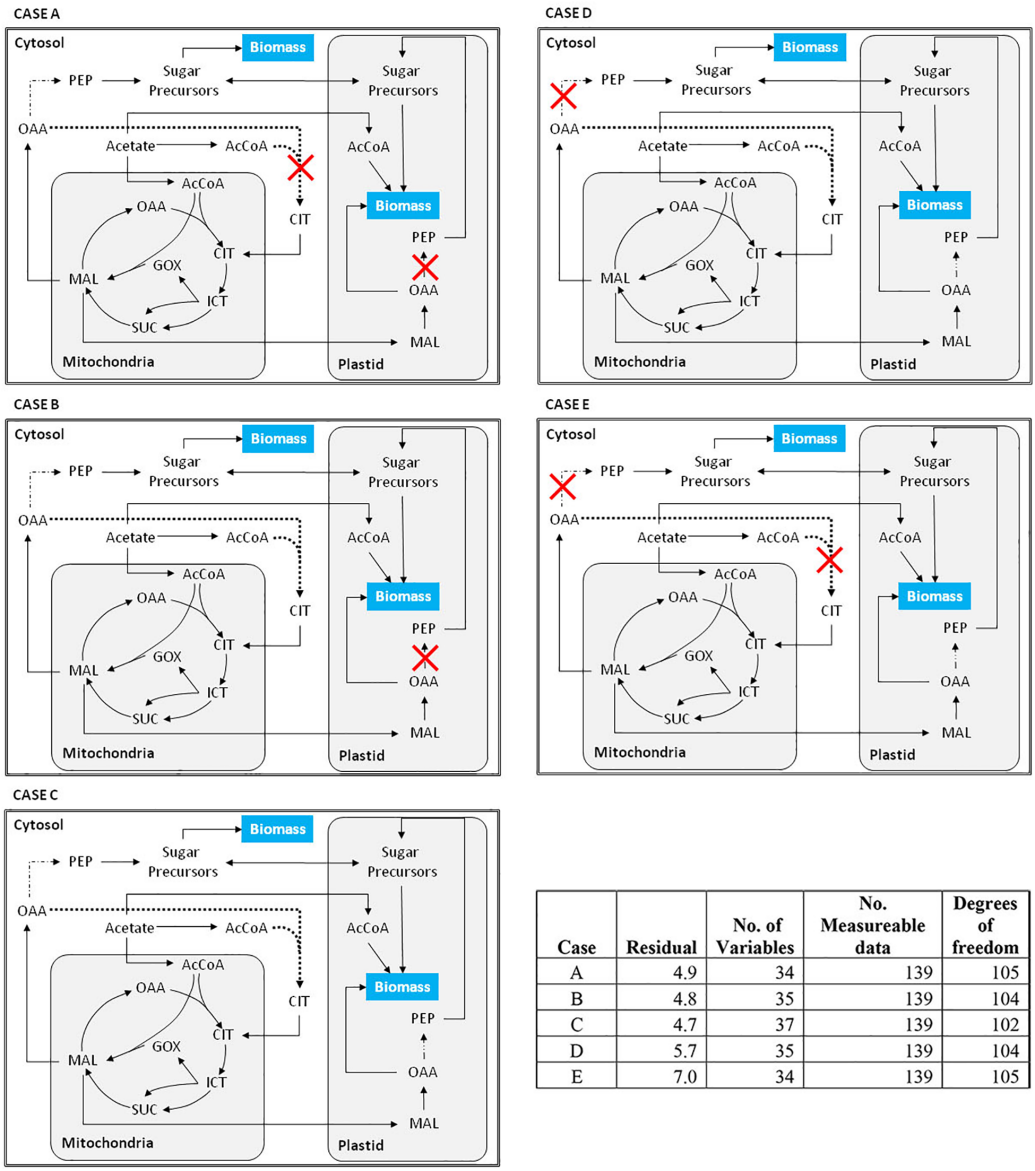
Simplified diagram depicting the different network cases studied. (A) The ‘base case’ in which the network only includes cytosolic gluconeogenesis. (B) This case, based on case A, also includes cytosolic citrate synthase, which was included based on genomic and proteomic evidence. (C) This case is case B with the addition of plastidic gluconeogensis. (D) No cytosolic gluconeogenesis was included, but cytosolic citrate synthase and plastidic gluconeogenesis are included. (E) Only plastidic gluconeogenesis is included. The table in the bottom right hand corner gives the residuals (calculated by F-statistic) and degrees of freedom for each case. Additional information about statistical significance is provided in supplemental info.

The ability to accurately model isotopic labeling patterns depends on the selected network topology and the fluxes through these networks. Compartment-specific labeling data was obtained based on the method developed by Allen et al. [31]. Different network topologies (i.e. the presence of a non-mitochondrial citrate synthase, and the role of cytosolic vs. plastidic gluconeogenesis) were evaluated by comparing residues by F-statistics [46] in order to determine which reactions should be included in the final network. The F-statistics were used as a basis of comparison to minimize effects of differing degrees of freedom between the different network topologies and to avoid overfitting (see methods). Fig 3 shows the different combinations of network topologies tested and Table 1 summarizes the residues for each case.

### Identification of metabolic pathways

#### Cytosolic gluconeogenesis

The importance of cytosolic gluconeogenesis was evaluated by comparing the network topologies where it was included or excluded. Case A and Case E differ in inclusion of cytosolic gluconeogenesis or its exclusion, respectively (Fig 3) and both lack the cytosolic citrate synthase. In comparing the statistical fits with the same degrees of freedom, we found the residual for Case A (4.9) was 30% less than Case E (7.0). Similarly, in Case B and Case D, which both have the cytosolic citrate synthase but differ in the presence or absence of cytosolic gluconeogenesis, Case B had a lower residual (4.8) than Case D (5.7) with the same degrees of freedom. As expected, it was apparent that including flux through cytosolic gluconeogenesis better matches the labeling pattern and therefore was selected.

#### Plastidic gluconeogenesis

In order to test whether plastidic gluconeogenesis should be included in the final network topology, we compared residuals between Case B and C (Fig 3). Inclusion of plastidic gluconeogenesis reactions (Case C) matched the labeling patterns with a slightly lower residue (4.74) when compared to Case B (4.76). However, the residual was not statistically different from Case B as determined by F-test due to the difference in the degrees of freedom. Therefore, the inclusion of plastidic gluconeogenesis cannot definitively be ruled out, but its inclusion did not significantly improve statistical fit to the data. The possibility that PCK1a was localized to mitochondria was also evaluated, but there was no statistical improvement in the fit to the data (data not shown).

#### Non-mitochondrial citrate synthase in acetate assimilation

Finally, the presence of a non-mitochondrial citrate synthase was also evaluated. The inclusion of a non-mitochondrial citrate synthase improved the statistical fit of the data in both Case B and D when compared to Case A and E with 94% and 99% confidence respectively. This provides strong evidence that a non-mitochondrial citrate synthase is indeed present and should be included in the final network topology.

#### Final network topology

Based on statistical analysis of possible network topologies derived from the Chlamydomonas genome, the optimal network topology which best describes heterotrophic growth was found to be Case B, and thus all subsequent simulations were based on this network topology.

### Isotopic dilution due to unlabeled biomass and CO_2_ from air

**Fig 4:**
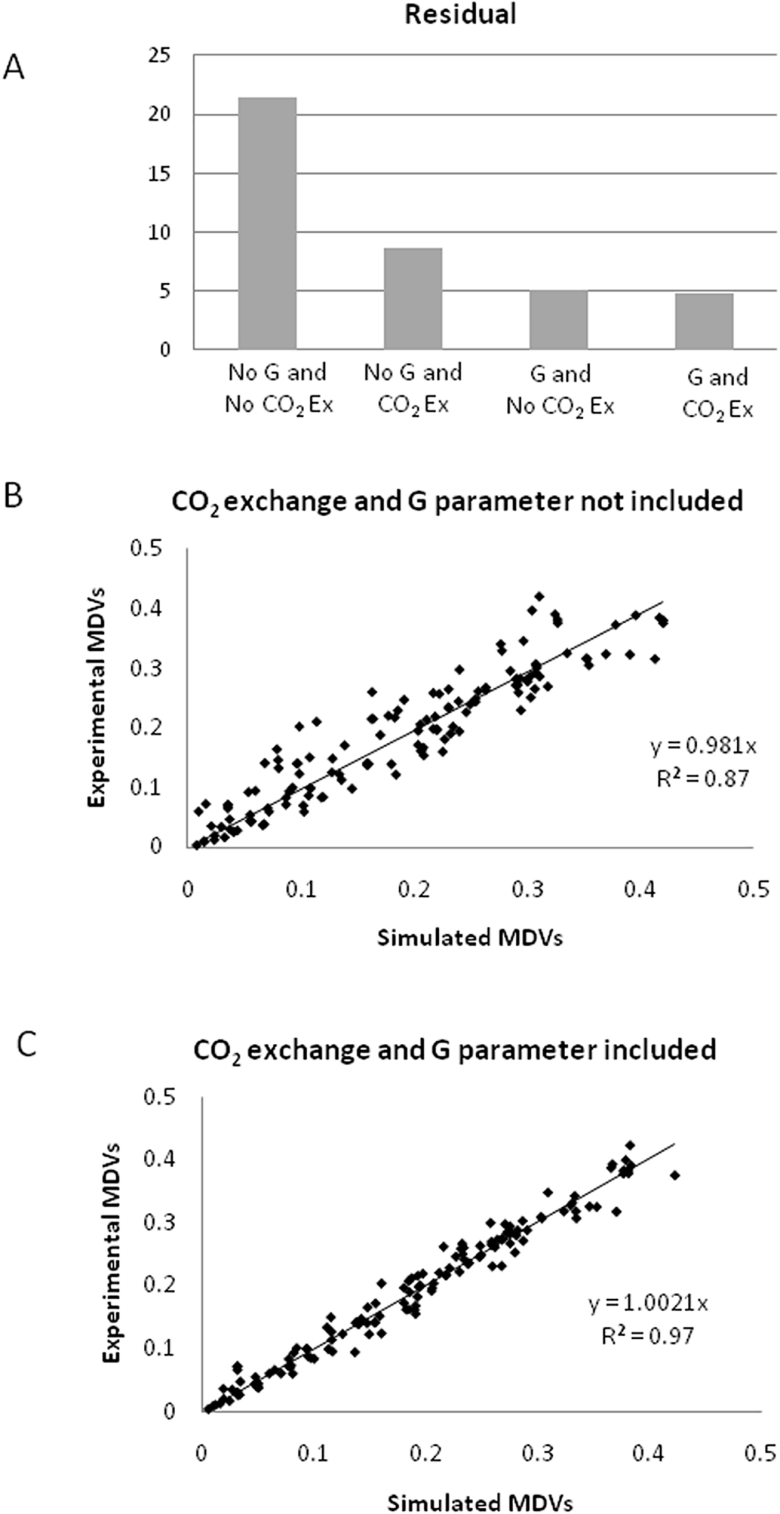
Effect of dilution due to CO_2_ and unlabeled biomass on the labeling patterns. (A) Comparison of residual when dilution due to preexisting biomass (G parameter), and dilution from CO_2_ in air was not included, and when either G or CO_2_ or both were included in the model; (B) Simulated vs. experimentally measured mass distribution vectors (MDVs) for the case when dilution due to CO_2_ and G parameter was not included in the model, (C) Simulated vs. experimentally measured MDVs for the case when dilution due to CO_2_ and G parameter included in the model. All plots correspond to the Case B, which has cytosolic citrate synthase and no plastidic gluconeogenesis.

Preliminary metabolic flux analysis simulations had high residual values in comparing the simulated against the experimental labeling patterns (Fig 4). In batch culture, one contributing factor for deviations in observed labeling patterns is the unlabeled biomass from the inoculum, which can be accounted for by the inclusion of the G parameter [45]. Another source for label dilution comes from unlabeled CO_2_ found in the sparging gas used in the reactor. During heterotrophic growth conditions, a net transport of CO_2_ out of the cell is expected due to respiration, however, CO_2_ can readily diffuse into the cell from the external media or can be actively transported into the cell [53]. For each case described above, we evaluated the impact of including the G parameter and CO_2_ exchange. As an example, Case B will be described. The base case simulation excluded the G parameter and CO_2_ exchange, a second simulation included CO_2_ exchange, which reduced the residual (Fig 4) by 60%. A third simulation included only the G parameter, which reduced the residual 76% compared to the base case; finally, the last simulation included both the G parameter and CO_2_ exchange which reduced the residual 78% percent. All the reported improvements in fit were significant at 99% statistical confidence, as determined by the F-test. It was also found that including both G parameter and dilution due to CO_2_ were significant at 99% confidence, when compared to only the CO_2_ dilution or G parameter included, respectively. Thus, incorporating both G parameter and dilution due to CO_2_ exchange greatly enhanced the ability to accurately fit experimental data. At the conclusion of pH-stat growth, the fraction of the original unlabeled biomass should be approximately 10% based upon growth measurements after isotopic labeling was initiated, which is close to the calculated dilution of 8% found in this study. This estimate was found by comparing the amount of biomass used for inoculation versus the amount of biomass resent when sampling (at 90 h). At this time point, the ratio of total biomass to the initial was approximately 9.5 which results in the % original biomass estimated at 10.5%. CO_2_ dilution from air had an exchange coefficient of 0.24 which corresponds to a dilution flux of 24% of the net outward CO_2_ flux.

### ^13^C flux map for Chlamydomonas under heterotrophic conditions

**Fig 5:**
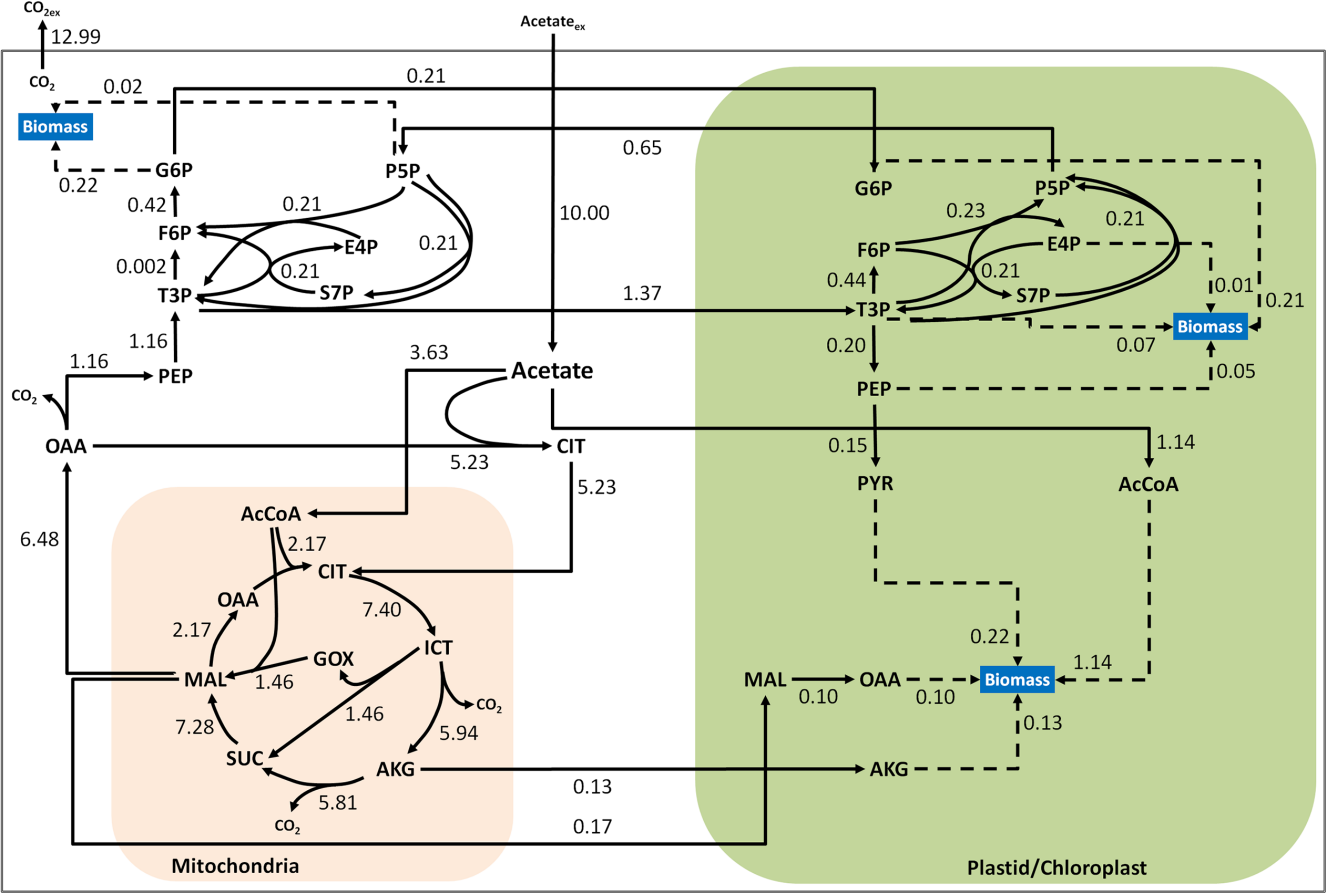
Fluxes calculated by ^13^C metabolic flux analysis for Case B (cytosolic citrate synthase and no plastidic gluconeogenesis). The fluxes are normalized to uptake rate of acetate on a basis of 10. The 90% confidence intervals for the fluxes are also reported in the supplementary materials (S7 Table).

The final flux map for acetate assimilation during heterotrophic growth conditions in *C*. *reinhardtii* is presented in Fig 5. This case gave us the best fit as judged from the lowest residuals and comparison of the various cases against each other by F-test to determine the statistical significance (Fig 4C). This model is based on Case B described above with the inclusion of both the G parameter (separate parameters for each component of biomass was included–protein, lipids, cell wall and sugars) and CO_2_ exchange. 95% confidence intervals for each flux are given in the supplementary material (S1 Table).

#### Utilization of acetate and tricarboxylic acid cycle fluxes

Based on the results of our ^13^C-MFA, we deduced that Chlamydomonas utilizes three pathways for acetate assimilation. A majority of the acetate taken up by the cell (53%) is converted to AcCoA by acetyl-coA synthetase in the cytoplasm and condensed with oxaloacetate (OAA) into citrate by citrate synthase before being transported across the mitochondrial membrane for entry into TCA cycle. The second pathway of acetate assimilation, the transport of acetate directly into the mitochondrion, accounts for roughly one-third of the total acetate uptake (35.6%). In the mitochondrion, acetate is converted to AcCoA and proceeds through the TCA cycle, providing reducing equivalents and biomass precursors. About 80% of the isocitrate flux in the mitochondrion is diverted through isocitrate dehydrogenase while 20% is sent through the glyoxylate shunt (isocitrate lyase), which indicates that a large portion of the flux is diverted towards NAD(P)H generation. The flux through mitochondrial malate dehydrogenase was 28% of the incoming flux into malate, indicating a minor contribution towards the TCA cycle. This is a direct effect of citrate being the major input into the TCA cycle. The majority, c.a. 72%, of the TCA cycle flux through malate is exported to the cytosol and converted to oxaloacetate. As shown in Fig 5 about 82% of the flux from cytosolic OAA is diverted towards the non-mitochondrial citrate synthase for acetate assimilation and to complete the TCA cycle, while the approximately 18% of the flux from oxaloacetate in the cytosol is directed towards gluconeogenesis.

#### Plastidic and cytoplasmic fluxes

All of the cytosolic gluconeogenesis flux is directed toward the production of cytosolic trioses; there is no flux through cytosolic pyruvate kinase and pyruvate dehydrogenase. Most of the flux through the trioses in the cytosol is directed toward the plastid. Overall, the fluxes in the plastid and cytoplasm derived from cytosolic gluconeogenesis account for roughly 11.5% of the acetate uptake flux. There is no evidence of oxidative pentose phosphate pathway flux in either the cytosol or plastid.

### Comparison of ^13^C-MFA fluxes to FBA predictions

**Fig 6:**
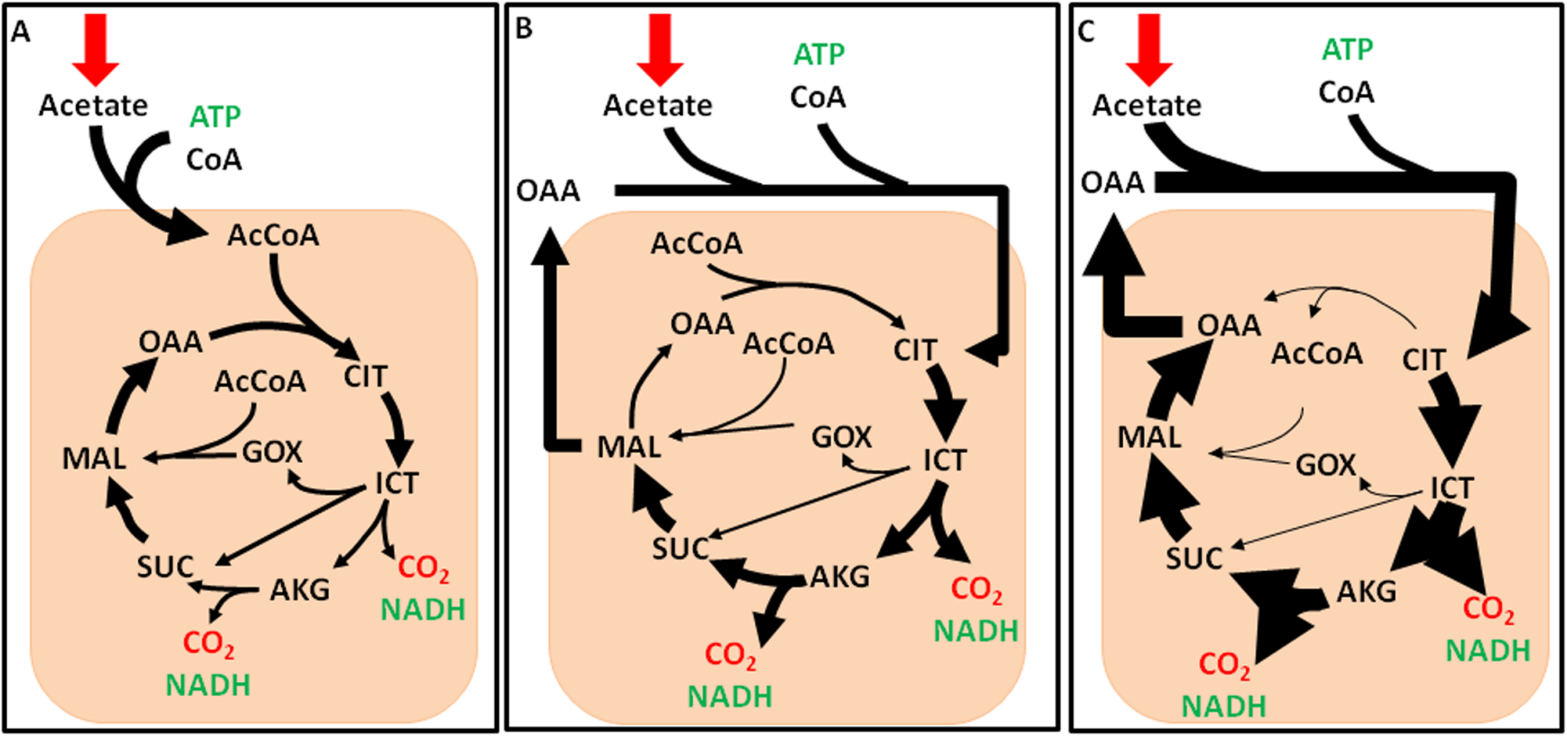
Comparison of TCA cycle fluxes calculated by flux balance analysis with metabolic flux analysis. (A) FBA fluxes with maximum biomass as the objective function. (B) ^13^C-MFA calculated fluxes. (C) FBA fluxes with maximum ATP production as the objective function. The thickness of the arrow in panels A-C depicts the amount of flux through the reaction.

Fluxes calculated by ^13^C-MFA were compared to predicted fluxes from flux balance analysis (FBA) for two different objective functions, maximize biomass and maximize ATP. There are two major differences between MFA and FBA; first, FBA includes balances on energy and reducing equivalents, and second, FBA uses linear programming to determine the optimal solution for the underdetermined system. In comparison, MFA uses experimental measurements to quantify *in vivo* fluxes. The FBA model originally reported by Boyle and Morgan [13] was used in its entirety for the simulations. In using the objective function ‘maximize biomass’, the model requires the cell to balance the need of substrate for biomass synthesis and the oxidation of substrate to produce energy in order to maximize biomass production. Due to our interest in acetate assimilation pathways, we choose to focus on the fluxes associated with the TCA cycle, shown in Fig 6A. In order to be carbon efficient, the model predicts 46% of the TCA cycle flux will be sent through the glyoxylate shunt, which bypasses two TCA cycle reactions that release carbon dioxide. In contrast to this, an objective function of maximum ATP forces the model to oxidize as much carbon as possible to produce ATP. This is shown in Fig 6C where almost all the carbon (96%) is directed through a complete TCA cycle; in this scheme, the cell loses 2 carbons for every turn of the cycle but also produces 2 NADH and 1 ATP. When these two conditions are compared to the *in vivo* fluxes calculated with ^13^C-MFA (Fig 6B), it appears that the actual objective function of the cell is a linear combination of both maximum biomass and maximum ATP.

## Discussion

### Glyoxylate shunt and TCA cycle

The glyoxlate shunt plays a key role in the utilization of acetate as it conserves the 2 carbons of acetate, and the flux through the shunt is indicative of acetate assimilated into biomass. In contrast, the flux through isocitrate dehydrogenase results in loss of CO_2_ and the production of NAD(P)H. We found that the split towards the glyoxylate shunt was only 20% of the incoming flux from isocitrate. By comparison, Samejima and Myers have reported 26% acetate assimilation to biomass in *Chlorella pyrenoidosa* in heterotrophic growth on acetate [54]. Flux analysis studies of *E*. *coli* and *S*. *cerevisiae* grown on acetate have a split towards glyoxylate of 47% and 27% of the total incoming isocitrate flux respectively [55, 56]. The relatively large flux towards isocitrate dehydrogenase observed in our case is indicative that the cell is producing copious amounts of NAD(P)H. The fate of this NAD(P)H was further investigated and it we found that approximately 28% would be necessary for biomass synthesis and gluconeogenesis, and the remainder could be utilized for ATP synthesis. Approximately 20% of the ATP demand was used for converting acetate to AcCoA. The demand for biomass synthesis from metabolite precursors and gluconeogenesis (calculated from the biomass synthesis equation) was found to be 21%. The major ATP demand was for maintenance energy and was 58% of the total ATP produced. Chen and Johns reported that inhibitory substrates such as acetate cause a large increase in the maintenance energy in Chlamydomonas [57, 58]. Thus in the present study we observe that the relatively large flux through isocitrate dehydrogenase is likely the result of a high maintenance energy requirement. A high level of ATP generation for cellular maintenance functions has also been reported in developing barley seeds [59] and for heterotrophic Arabidopsis cell cultures [60].

### The role of non-mitochondrial citrate synthase

We evaluated several different network topologies based on the presence of specific genes in the Chlamydomonas genome using ^13^C-MFA to determine the best fit to our data. This analysis resulted in improved fit with the inclusion of a cytosolic citrate synthase, indicating that there is indeed activity of non-mitochondrial citrate synthase in heterotrophically grown Chlamydomonas, which has been reported previously [50]. It is difficult to categorically state which citrate synthase, the cytosolic or mitochondrial form, plays a larger role in acetate assimilation in Chlamydomonas because of the relatively large error bounds for these two reactions from ^13^C-MFA. This problem could be addressed by obtaining more detailed compartment specific isotopic labeling data; however, the premier method used in plant cells, non-aqueous fractionation [61, 62], has yet to be reported in any algae species.

### Plastidic fluxes

**Fig 7:**
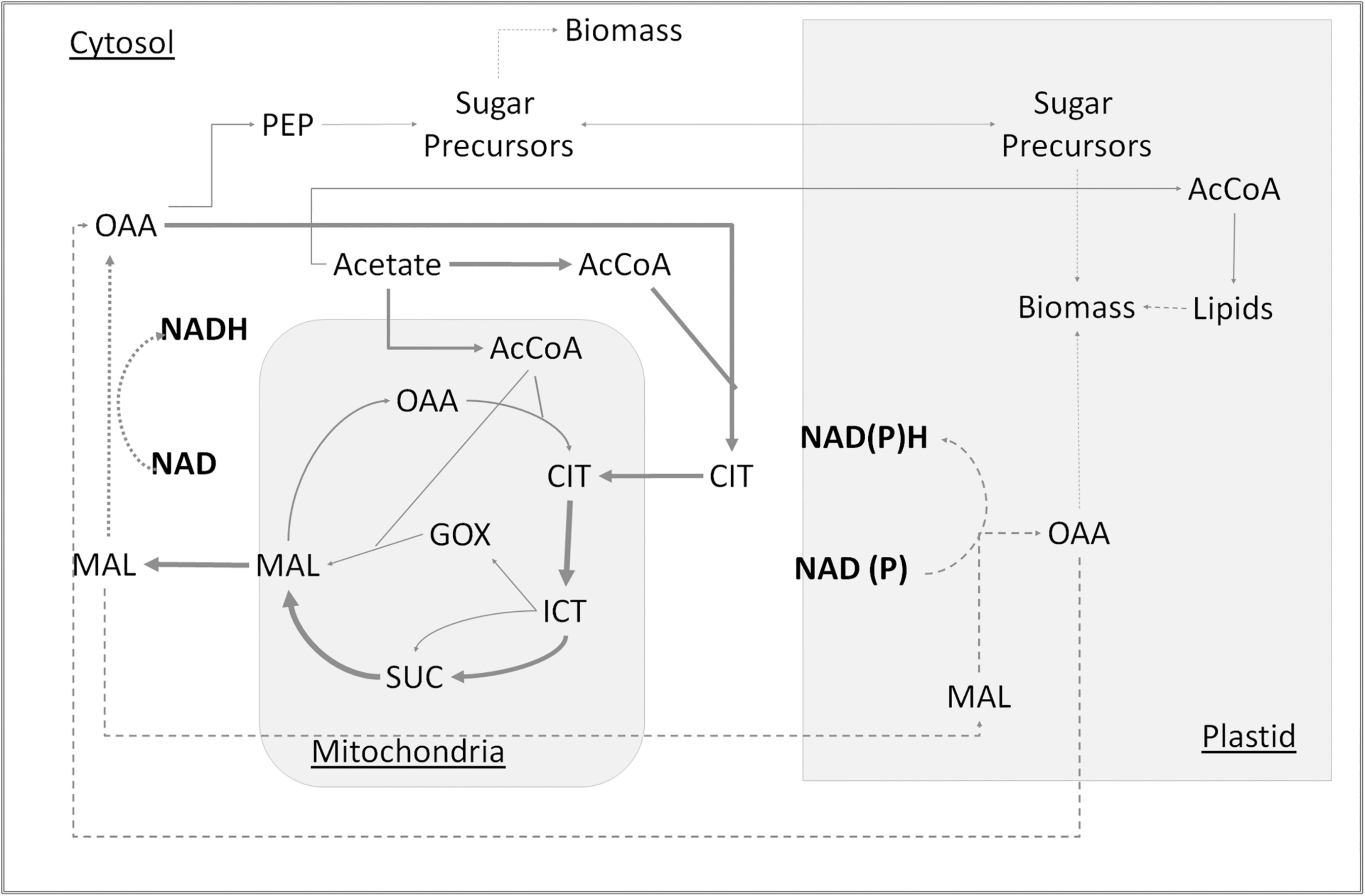
Simplified flux map depicting reducing equivalent shuttling to plastid for network case B. A flux map where a part of the malate is shuttled to cytosol (shown by dotted arrow) and rest is shuttled towards plastid for transferring reducing equivalents to plastid (shown by dash arrows). The resulting oxaloacetate in plastid is shuttled back to the cytosol for utilization by citrate synthase. The arrow thickness corresponds to actual fluxes and malate split towards plastid is estimated to be 35% of the malate flux into cytosol from mitochondria.

In our MFA calculations, no flux through the oxidative pentose phosphate pathway (oxPPP) was found in the plastid. Low plastidic oxPPP was also observed in heterotrophic growth on glucose for Arabidopsis suspension culture [32]. Many biosynthetic reactions occur in the plastid, which need reducing equivalents. Hence reducing equivalents NADPH/NADH must be generated from another pathway, within the plastid or translocated from the cytosol. One possible way is to transport a portion of the malate flux to the plastid and then convert it to oxaloacetate for generating the required reducing equivalents in the plastid. Plastidic oxaloacetate can then be transported back to cytosol, where it is recycled back into the TCA cycle via cytosolic citrate synthase (Fig 7). Redox independent plastidic malate dehydrogenase (MDH-NADH) has been shown to play a critical role in heterotrophic growth in developing seed of *Arabidopis* [63], where a t-DNA mutant had a severe growth restriction. Concomitant increase in expression of levels of an alternative NAD+-regenerating enzyme, NADH–GOGAT was also observed. A probable shuttle mechanism for this type of transport into chloroplast would be malate-asparate type shuttle for chloroplast; a chloroplastic oxoglutarate:malate translocater (OMT1) has been reported in *C*. *reinhardtii* [40]. It should be noted that it is not possible to distinguish by isotopic labeling alone if the cytosolic oxaloacetate comes directly from malate in the mitochondrion (Fig 7) or if a part of it comes from malate in the plastid recycle as shown in Fig 7. The flux split around malate was calculated based on NAD(P)H demand for fatty acid synthesis in plastids. The total reducing equivalents required for gluconeogenesis and fatty acid synthesis combined was only 72% of that produced in the cytosol. Thus, if there are additional NAD(P)H demands in plastid and cytosol, it would affect the actual split of malate towards cytosol and plastid. Alternatively, the shuttling of the reducing equivalents could involve transport of cytosolic, Glyceraldehyde-3-phosphate (G3P) into plastid. G3P would be formed from reduction and phosphorylation of 3-phosphoglycerate (3PG), formed from Phosphoenolpyruvate (PEP), which in turn would be formed from cytosolic gluconeogenesis. The translocated G3P can be converted back to 3PG, and translocated back to cytosol, hence providing the reducing equivalents to the plastid [64]. In this study, a net transport of cytosolic T3P was observed towards plastid. Separate 3PG and G3P pools were not included, as they are indistinguishable based on the measurements.

The overall fluxes in the plastid were relatively low and were mainly for providing precursors for biomass. The main carbon source for plastidic biomass synthesis, aside from lipids, was triose phosphates imported from the cytosol. Interestingly, we found that plastidic fatty acid synthesis occurs via direct assimilation of acetate with no apparent contribution from plastidic pyruvate. The direct assimilation of acetate to lipids was also observed in *S*. *cerevisiae* grown on acetate [56]. Thus, under heterotrophic conditions in *C*. *reinhardtii*, there is no direct competition between fatty acid synthesis and starch synthesis. A similar result was reported by Li et al. for triacylglycerol (TAG) production in *C*. *reinhardtii;* when grown heterotrophically, there was no significant difference in the total amount of TAG between a starchless mutant and its parent strain [65]. Labelling data indicates that malic enzyme (MME4) is not active, which has also been reported in *C*. *protothecoides* for growth on glucose [19, 37]. We also evaluated the possibility of plastidic and mitochondrial gluconeogenesis (in addition to cytosolic gluconeogenesis), and found that inclusion of these pathways produced no statistical improvement in the model. However, addition of these alternative routes could not be ruled out by ^13^C labeling data alone. Thus, we could not definitively distinguish whether plastidic gluconeogenesis in addition to the cytosolic gluconeogenesis is actually playing a physiological role in heterotrophic growth.

### FBA predictions

Fluxes obtained from ^13^C-MFA were compared with fluxes predicted using FBA with either maximal growth or for maximal ATP production as objective functions. Although fluxes are readily estimated for a variety of objective functions, they do not necessarily reflect what is actually occurring in the cell, if the cell metabolism is suboptimal. Previous studies of *E*. *coli* grown on acetate or succinate have shown that FBA can predict intracellular fluxes quite well with an objective function of maximizing biomass [66]. This implies that *E*. *coli* grown under these conditions operates at near optimal conditions. While the two objective functions we evaluated are in no way an exhaustive search of the solution space, they are two of the most widely used in trying to capture wild type growth. In this study, FBA is not able to accurately capture intracellular fluxes (Fig 6) quantitatively or qualitatively by either of the objective functions tested here, similar to a study on *B*. *subtilis* [21]. In comparing the two objective functions for their ability to predict fluxes in *C*. *reinhardtii*, neither captures the *in vivo* fluxes, however maximizing ATP captures the qualitative aspects of the fluxes. This implies that the heterotrophic growth of *C*. *reinhardtii* is actually suboptimal and that the actual cellular objective of the cell cannot be captured simply by a single objective function. The higher flux through the TCA cycle as determined from ^13^C-MFA shows that *C*. *reinhardtii* oxidizes a significant portion of carbon for the production of ATP, which likely is caused by high maintenance demands. Changes in carbon use efficiency were also observed under stress conditions in Arabidopsis cell cultures [60]. Under suboptimal growth conditions, it becomes clear that MFA is a powerful technique to accurately capture the *in vivo* carbon fluxes in *C*. *reinhardtii*.

## Conclusion

Using ^13^C-labeling and flux analysis, fluxes for *C*. *reinhardtii* under heterotrophic growth on acetate were determined. We found that the first steps of acetate assimilation were distributed among all three intracellular compartments. In particular, we found that a non-mitochondrial citrate synthase (modeled in the cytoplasm) plays an important role in acetate assimilation based on the reduced residuals when included in the network topology. However, we are unable to definitively determine the relative contribution of the cytosolic form over the mitochondrial form. These fluxes could potentially be resolved with more detailed compartment specific isotope labeling data, or improved methods for fast and specific subcellular fractionation in algae. The remaining acetate is assimilated directly into fatty acids in the plastid and is determined mainly by the demand for lipids in the biomass composition. Fluxes obtained from our ^13^C-MFA experiment were compared to predictions made by FBA. We found that neither objective function of maximum biomass or maximum ATP could quantitatively predict intracellular fluxes; in fact, *C*. *reinhardtii* grew suboptimally, sacrificing carbon for energy production. Metabolic flux analysis using isotopically labeled substrates can be further used for studying different growth modes, such as autotrophic or mixotrophic growth with consideration of dynamic labeling patterns [67, 68], which is of particular interest for the production of biodiesel in algae due to the higher TAG contents reported in mixotrophic cultures [65]. An interesting application of this technique in future studies would be to investigate fluxes during macronutrient starvation, due to the current interest in algae biofuels and recent reports of *C*. *reinhardtii* accumulating lipids when starved for nitrogen [69–72], sulfur [73] or trace nutrients [74].

## Supporting information

S1 Fig Heterotrophic growth of *Chlamydomonas reinhardtii* in pH-stat mode in bioreactor. Cells were sampled in mid-exponential phase as is evident in the plot of ln(OD/OD_o_) (panel A). Stationary phase is typically not reached until approximately 130 hours. pH in the bioreactor during the course of the experiment is shown in panel B.
(DOCX)

S2 Fig **Statistical evaluation of the residuals for Case B:** (A) Probability plot for residuals approximated by a normal distribution of μ = -.0774 and σ = 1.85, indicated that the residuals were normally distributed. (B) Residuals versus Mass distribution vectors (MDV) indicated no correlation.
(DOCX)

S3 Fig Isotopomer modeling strategy. For a given initial estimate for fluxes, the set of isotopomer balances were solved to obtain isotopomer distribution vectors (IDV). The isotopomer distribution vectors (IDV) were used for calculation of the Mass distribution vectors (MDV), and the calculated MDVs were used for least square optimization. The objective function was minimization of the sum of the squares of the difference between the experimentally observed and simulated MDVs. The iterative process is repeated till a minimum is found to obtain a set of fluxes that can explain the labeling patterns[26, 27, 43, 44].
(DOCX)

S1 Table Estimated normalized flux values for Case B and confidence bounds at 95% confidence interval. Flux values in mol/10 mol of acetate uptaken.
(DOCX)

S2 Table Experimental measurements for heterotrophic growth of *C*. *reinhardtii*. (DOCX)

S3 Table Isotope distribution of cell wall derived sugars in heterotrophic grown *C*. *reinhardtii*. (DOCX)

S4 Table Isotope distribution of starch derived sugars in heterotrophic *C*. *reinhardtii*. (DOCX)

S5 Table Isotope distribution of amino acids in heterotrophic *C*. *reinhardtii*. (DOCX)

S6 Table Isotope distribution of additional amino acid fragments as described by Antoniewicz et al. (DOCX)

S7 Table Additional Isotope distribution of TMS derivatives of sugar fragments as described in MacLeod et al. (DOCX)

S8 Table Coefficients for biomass formation equation used in ^13^C-MFA and FBA calculations given in moles per kilogram biomass. OA and MAL are lumped, as are PEP and 3PG.
(DOCX)

S9 Table Estimated normalized net fluxes, exchange coefficients and G parameters for Case B with standard deviation(s.d.), estimated from 95% intervals. Flux values in mol/10 mol of acetate.
(DOCX)

S10 Table The calculated F-statistic and statistical significance from comparing the different network topologies. (DOCX)
