# Supplementary material for: Metabolic flux analysis of heterotrophic growth in Chlamydomonas reinhardtii
Source: PLoS One. 2017 May 24;12(5):e0177292. doi: 10.1371/journal.pone.0177292 (PMC5443493; doi:10.1371/journal.pone.0177292)
Supplement: S1 Fig — Cells were sampled in mid-exponential phase as is evident in the plot of ln(OD/ODo) (panel A). Stationary phase is typically not reached until approximately 130 hours. pH in the bioreactor during the course of the experiment is shown in panel B. (DOCX) [file pone.0177292.s001.docx]

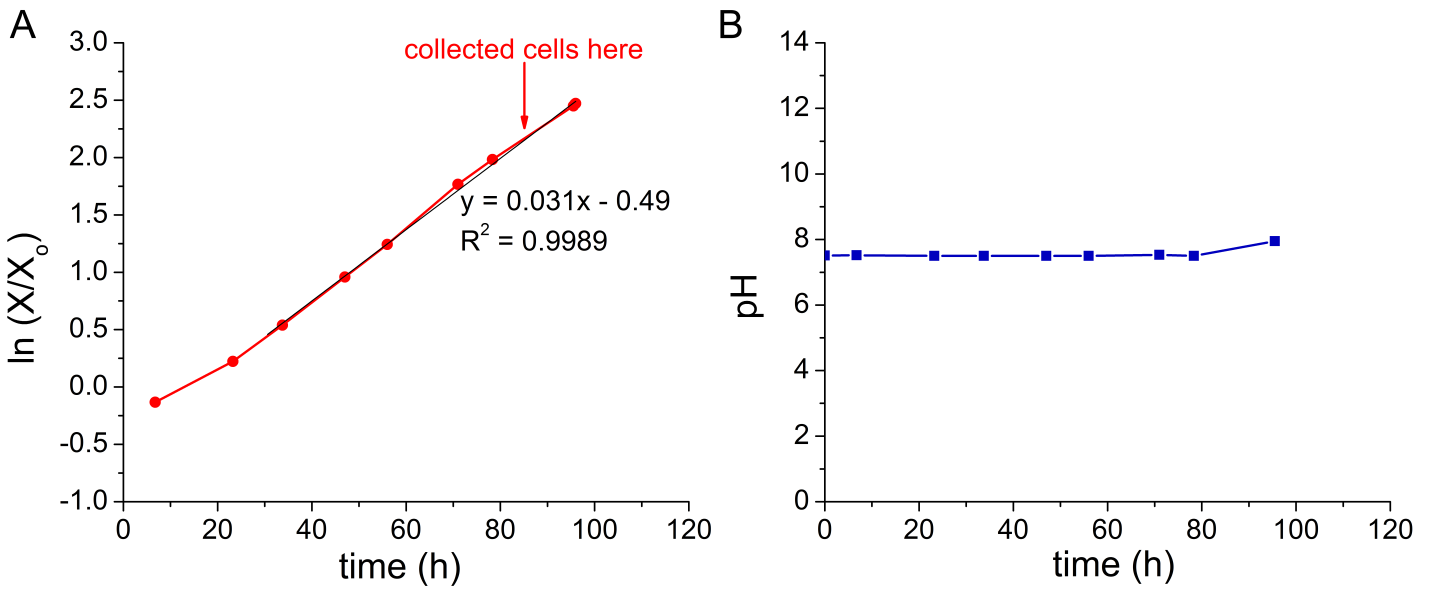


S1 Fig. Heterotrophic growth of *Chlamydomonas reinhardtii* in pH-stat mode in bioreactor. Cells were sampled in mid-exponential phase as is evident in the plot of ln(OD/OD_o_) (panel A). Stationary phase is typically not reached until approximately 130 hours. pH in the bioreactor during the course of the experiment is shown in panel B.
