# Supplementary material for: Metabolic flux analysis of heterotrophic growth in Chlamydomonas reinhardtii
Source: PLoS One. 2017 May 24;12(5):e0177292. doi: 10.1371/journal.pone.0177292 (PMC5443493; doi:10.1371/journal.pone.0177292)
Supplement: S2 Fig — Statistical evaluation of the residuals for Case B: (A) Probability plot for residuals approximated by a normal distribution of μ = -.0774 and σ = 1.85, indicated that the residuals were normally distributed. (B) Residuals versus Mass distribution vectors (MDV) indicated no correlation. (DOCX) [file pone.0177292.s002.docx]

**A**

**B**


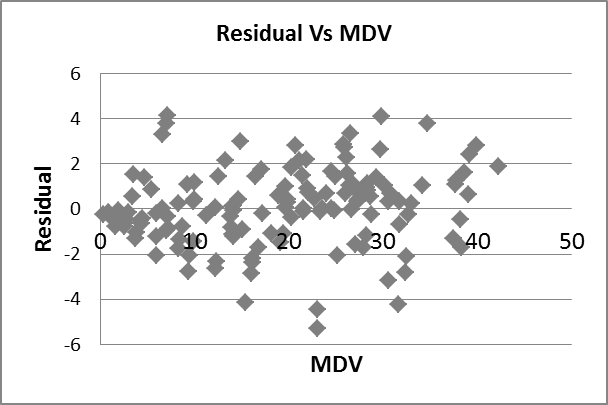


**S2 Fig. Statistical evaluation of the residuals for Case B: (**A) Probability plot for residuals approximated by a normal distribution of µ = -.0774 and σ = 1.85, indicated that the residuals were normally distributed. (B) Residuals versus Mass distribution vectors (MDV) indicated no correlation.
