# Supplementary material for: Metabolic flux analysis of heterotrophic growth in Chlamydomonas reinhardtii
Source: PLoS One. 2017 May 24;12(5):e0177292. doi: 10.1371/journal.pone.0177292 (PMC5443493; doi:10.1371/journal.pone.0177292)
Supplement: S3 Fig — For a given initial estimate for fluxes, the set of isotopomer balances were solved to obtain isotopomer distribution vectors (IDV). The isotopomer distribution vectors (IDV) were used for calculation of the Mass distribution vectors (MDV), and the calculated MDVs were used for least square optimization. The objective function was minimization of the sum of the squares of the difference between the experimentally observed and simulated MDVs. The iterative process is repeated till a minimum is found to obtain a set of fluxes that can explain the labeling patterns[26, 27, 43, 44]. (DOCX) [file pone.0177292.s003.docx]

**S3 Fig. Isotopomer modeling strategy**. For a given initial estimate for fluxes, the set of isotopomer balances were solved to obtain isotopomer distribution vectors (IDV). The isotopomer distribution vectors (IDV) were used for calculation of the Mass distribution vectors (MDV), and the calculated MDVs were used for least square optimization. The objective function was minimization of the sum of the squares of the difference between the experimentally observed and simulated MDVs. The iterative process is repeated till a minimum is found to obtain a set of fluxes that can explain the labeling patterns. [[1-4](#_ENREF_1)]

**References**

1. Zupke C, Stephanopoulos G. Modeling of Isotope Distributions and Intracellular Fluxes in Metabolic Networks Using Atom Mapping Matrixes. Biotechnology Progress. 1994;10(5):489-98. doi: 10.1021/bp00029a006.

2. Schmidt K, Carlsen M, Nielsen J, Villadsen J. Modeling isotopomer distributions in biochemical networks using isotopomer mapping matrices. Biotechnology and Bioengineering. 1997;55(6):831-40. doi: 10.1002/(sici)1097-0290(19970920)55:6<831::aid-bit2>3.0.co;2-h.

3. Schmidt K, Nielsen J, Villadsen J. Quantitative analysis of metabolic fluxes in *Escherichia coli*, using two-dimensional NMR spectroscopy and complete isotopomer models. Journal of Biotechnology. 1999;71(1-3):175-89.

4. Wiechert W, Möllney M, Petersen S, de Graaf AA. A Universal Framework for ^13^C Metabolic Flux Analysis. Metabolic Engineering. 2001;3(3):265-83. doi: DOI: 10.1006/mben.2001.0188.
