# Supplementary material for: Metabolic flux analysis of heterotrophic growth in Chlamydomonas reinhardtii
Source: PLoS One. 2017 May 24;12(5):e0177292. doi: 10.1371/journal.pone.0177292 (PMC5443493; doi:10.1371/journal.pone.0177292)
Supplement: S1 Table — Flux values in mol/10 mol of acetate uptaken. (DOCX) [file pone.0177292.s004.docx]

**S1 Table. Estimated normalized flux values for Case B and confidence bounds at 95% confidence interval.** **Flux values in mol/10 mol of acetate uptaken**

| **Flux** | **Lower Bound** | **Calculated Flux** | **Upper Bound** | **Flux** | **Lower Bound** | **Calculated Flux** | **Upper Bound** | **Flux** | **Lower Bound** | **Calculated Flux** | **Upper Bound** |
| --- | --- | --- | --- | --- | --- | --- | --- | --- | --- | --- | --- |
| v1 | 0.00 | 0.00 | 0.18 | v28 | 7.18 | 47.28 | 47.27 | v55 | 0.00 | 0.17 | 0.22 |
| v2 | 0.01 | 1.16 | 38.05 | v29 | 0.00 | 12.98 | 39.74 | v56 | 0.00 | 0.00 | 0.44 |
| v3 | 0.02 | 1.58 | 38.29 | v30 | 2.10 | 15.08 | 41.83 | v57 | 0.02 | 0.02 | 0.02 |
| v4 | 0.00 | 0.00 | 3.78 | v31 | 0.15 | 34.75 | 40.76 | v58 | 0.22 | 0.22 | 0.22 |
| v5 | 0.00 | 0.00 | 2.73 | v32 | 0.00 | 34.60 | 40.00 | v59 | 0.21 | 0.21 | 0.21 |
| v6 | 0.29 | 40.21 | 40.44 | v33 | 5.75 | 11.49 | 46.41 | v60 | 0.01 | 0.01 | 0.01 |
| v7 | 0.00 | 40.00 | 40.00 | v34 | 0.00 | 0.05 | 0.66 | v61 | 0.07 | 0.07 | 0.07 |
| v8 | 0.00 | 10.67 | 40.00 | v35 | 1.00 | 1.01 | 1.36 | v62 | 0.05 | 0.05 | 0.05 |
| v9 | 0.29 | 10.87 | 40.31 | v36 | 0.55 | 15.50 | 40.00 | v63 | 1.14 | 1.14 | 1.14 |
| v10 | 0.00 | 0.67 | 38.99 | v37 | 0.00 | 15.30 | 40.13 | v64 | 0.10 | 0.10 | 0.10 |
| v11 | 0.01 | 0.46 | 38.71 | v38 | 0.00 | 0.00 | 15.23 | v65 | 0.13 | 0.13 | 0.13 |
| v12 | 0.00 | 40.00 | 40.00 | v39 | 0.00 | 0.65 | 15.63 | v66 | 0.22 | 0.22 | 0.22 |
| v13 | 1.27 | 41.18 | 41.31 | v40 | 0.02 | 1.38 | 17.77 | v67 | 0.00 | 0.00 | 0.00 |
| v14 | 1.24 | 41.18 | 41.31 | v41 | 0.00 | 0.00 | 16.85 | v68 | 0.70 | 1.14 | 1.14 |
| v15 | 0.00 | 0.00 | 0.19 | v42 | 0.00 | 0.00 | 40.00 | v69 | 10.00 | 10.00 | 10.00 |
| v16 | 0.00 | 0.00 | 0.19 | v43 | 0.01 | 0.00 | 41.47 | v70 | 0.00 | 5.30 | 8.86 |
| v17 | 0.00 | 3.56 | 8.86 | v44 | 0.00 | 0.00 | 0.37 | v71 | 0.00 | 0.58 | 189.00 |
| v18 | 0.44 | 2.68 | 191.13 | v45 | 0.44 | 32.89 | 40.55 | v72 | 166.43 | 166.43 | 166.43 |
| v19 | 1.46 | 1.46 | 1.90 | v46 | 0.00 | 32.45 | 40.00 | v73 | 0.00 | 0.00 | 0.00 |
| v20 | 7.28 | 7.40 | 7.40 | v47 | 0.22 | 33.73 | 40.23 | v74 | 0.00 | 0.00 | 0.00 |
| v21 | 0.00 | 0.00 | 0.00 | v48 | 0.00 | 33.50 | 40.00 | v75 | 0.90 | 0.91 | 1.26 |
| v22 | 5.50 | 5.94 | 5.94 | v49 | 0.00 | 0.21 | 39.94 | v76 | 0.00 | 40.00 | 40.00 |
| v23 | 0.13 | 0.13 | 0.13 | v50 | 0.00 | 0.00 | 39.95 | v77 | 0.00 | 5.01 | 39.95 |
| v24 | 0.00 | 0.00 | 0.00 | v51 | 0.00 | 0.23 | 29.35 | v78 | 0.00 | 0.00 | 0.00 |
| v25 | 5.38 | 5.82 | 5.82 | v52 | 0.00 | 0.02 | 29.34 | v79 | 12.99 | 12.99 | 12.99 |
| v26 | 1.46 | 1.46 | 1.90 | v53 | 0.06 | 0.59 | 37.01 | v80 | 14.17 | 14.43 | 15.93 |
| v27 | 0.00 | 40.00 | 40.00 | v54 | 0.00 | 0.38 | 36.81 | v81 | 1.18 | 1.44 | 2.94 |
