# Supplementary material for: Metabolic flux analysis of heterotrophic growth in Chlamydomonas reinhardtii
Source: PLoS One. 2017 May 24;12(5):e0177292. doi: 10.1371/journal.pone.0177292 (PMC5443493; doi:10.1371/journal.pone.0177292)
Supplement: S2 Table — (DOCX) [file pone.0177292.s005.docx]

**S2 Table. Experimental measurements for heterotrophic growth of *C. reinhardtii*.**

| **Biomass Yield (g/mole acetate)** | 16.60 |
| --- | --- |
| **Lipid Fraction (g/g DW)** | 0.290 |
| **Growth Rate (hr^-1^)** | 0.030 |
