# Supplementary material for: Metabolic flux analysis of heterotrophic growth in Chlamydomonas reinhardtii
Source: PLoS One. 2017 May 24;12(5):e0177292. doi: 10.1371/journal.pone.0177292 (PMC5443493; doi:10.1371/journal.pone.0177292)
Supplement: S3 Table — (DOCX) [file pone.0177292.s006.docx]

**S3 Table. Isotope distribution of cell wall derived sugars in heterotrophic grown *C. reinhardtii***

| **Cell Wall Sugars** | **Ion** | **M+0** | **M+1** | **M+2** | **M+3** | **M+4** | **M+5** |
| --- | --- | --- | --- | --- | --- | --- | --- |
| Glucose | 319 | 0.2190 | 0.2544 | 0.2644 | 0.1676 | 0.0724 | 0.0222 |
|  | 160 | 0.3607 | 0.3841 | 0.1823 | 0.0729 |  |  |
| Galactose | 319 | 0.2030 | 0.2641 | 0.2664 | 0.1721 | 0.0718 | 0.0225 |
|  | 160 | 0.3833 | 0.3773 | 0.1676 | 0.0718 |  |  |
| Mannose | 319 | 0.5750 | 0.2164 | 0.1351 | 0.0494 | 0.0184 | 0.0058 |
|  | 160 | 0.6229 | 0.2354 | 0.0872 | 0.0544 |  |  |
| Xylose | 217 | 0.2301 | 0.3175 | 0.2603 | 0.1259 | 0.0518 | 0.0144 |
|  | 160 | 0.3162 | 0.3643 | 0.1877 | 0.1317 |  |  |
| Arabinose | 217 | 0.2492 | 0.3217 | 0.2603 | 0.1091 | 0.0475 | 0.0122 |
|  | 160 | 0.3025 | 0.3734 | 0.1981 | 0.1260 |  |  |
