# Supplementary material for: Metabolic flux analysis of heterotrophic growth in Chlamydomonas reinhardtii
Source: PLoS One. 2017 May 24;12(5):e0177292. doi: 10.1371/journal.pone.0177292 (PMC5443493; doi:10.1371/journal.pone.0177292)
Supplement: S4 Table — (DOCX) [file pone.0177292.s007.docx]

**S4 Table. Isotope distribution of starch derived sugars in heterotrophic *C. reinhardtii.***

| **Starch Sugars** | **Ion** | **M+0** | **M+1** | **M+2** | **M+3** | **M+4** | **M+5** | **M+6** | **M+7** |
| --- | --- | --- | --- | --- | --- | --- | --- | --- | --- |
| Glucose | 319 | 0.1237 | 0.2688 | 0.2936 | 0.1962 | 0.0831 | 0.0264 | 0.0070 | 0.0013 |
|  | 160 | 0.3777 | 0.3822 | 0.1546 | 0.0657 | 0.0153 | 0.0045 |  |  |
| Galactose | 319 | 0.1244 | 0.2696 | 0.2940 | 0.1952 | 0.0825 | 0.0260 | 0.0069 | 0.0014 |
|  | 160 | 0.1038 | 0.1031 | 0.0410 | 0.0147 | 0.0035 | 0.0009 |  |  |
