# Supplementary material for: Metabolic flux analysis of heterotrophic growth in Chlamydomonas reinhardtii
Source: PLoS One. 2017 May 24;12(5):e0177292. doi: 10.1371/journal.pone.0177292 (PMC5443493; doi:10.1371/journal.pone.0177292)
Supplement: S5 Table — (DOCX) [file pone.0177292.s008.docx]

**S5 Table. Isotope distribution of amino acids in heterotrophic *C. reinhardtii.***

| **Amino Acids** | **Fragments** | **Ion**  **(m/z)** | **M+0** | **M+1** | **M+2** | **M+3** | **M+4** | **M+5** | **M+6** | **M+7** | **M+8** | **M+9** | **M+10** | **M+11** | **M+12** |
| --- | --- | --- | --- | --- | --- | --- | --- | --- | --- | --- | --- | --- | --- | --- | --- |
| Alanine | *C-1-2-3* | 260 | 0.2470 | 0.3273 | 0.2722 | 0.1153 | 0.0307 | 0.0075 |  |  |  |  |  |  |  |
| Glycine | *C-1-2* | 246 | 0.3521 | 0.3313 | 0.2398 | 0.0588 | 0.0181 |  |  |  |  |  |  |  |  |
| Valine | *C-1-2-3-4-5* | 288 | 0.1417 | 0.2596 | 0.2872 | 0.1950 | 0.0845 | 0.0258 | 0.0061 |  |  |  |  |  |  |
| Leucine | *C-2-3-4-5-6* | 274 | 0.1649 | 0.2988 | 0.2874 | 0.1607 | 0.0597 | 0.0166 | 0.0033 | 0.0057 | 0.0016 |  |  |  |  |
| Isoleucine | *C-2-3-4-5-6* | 274 | 0.1467 | 0.2580 | 0.2839 | 0.1910 | 0.0836 | 0.0262 | 0.0059 | 0.0046 |  |  |  |  |  |
| Serine | *C-1-2-3* | 390 | 0.2373 | 0.3164 | 0.2628 | 0.1266 | 0.0435 | 0.0107 | 0.0027 |  |  |  |  |  |  |
| Threonine | *C-2-3-4* | 376 | 0.1411 | 0.2338 | 0.2838 | 0.1989 | 0.0997 | 0.0331 | 0.0095 |  |  |  |  |  |  |
| Phenylalanine | *C-1-2-3-4-5-6-7-8-9* | 336 | 0.0602 | 0.0927 | 0.1713 | 0.2199 | 0.2029 | 0.1398 | 0.0701 | 0.0282 | 0.0090 | 0.0027 | 0.0015 | 0.0011 | 0.0006 |
| Aspartate | *C-1-2-3-4* | 418 | 0.1383 | 0.2341 | 0.2827 | 0.1987 | 0.1006 | 0.0335 | 0.0099 | 0.0022 |  |  |  |  |  |
| Glutamate | *C-1-2-3-4-5* | 432 | 0.0949 | 0.1889 | 0.2625 | 0.2273 | 0.1406 | 0.0599 | 0.0195 | 0.0053 | 0.0011 |  |  |  |  |
