# Supplementary material for: Metabolic flux analysis of heterotrophic growth in Chlamydomonas reinhardtii
Source: PLoS One. 2017 May 24;12(5):e0177292. doi: 10.1371/journal.pone.0177292 (PMC5443493; doi:10.1371/journal.pone.0177292)
Supplement: S6 Table — (DOCX) [file pone.0177292.s009.docx]

**S6 Table. Isotope distribution of additional amino acid fragments as described by Antoniewicz et al. [**[**1**](#_ENREF_1)**].**

| **Amino Acid** | **Fragment** | **Mass** | **M+0** | **M+1** | **M+2** | **M+3** | **M+4** | **M+5** | **M+6** | **M+7** | **M+8** |
| --- | --- | --- | --- | --- | --- | --- | --- | --- | --- | --- | --- |
| Threonine | *C-1-2-3-4* | 404 | 0.2161 | 0.3083 | 0.2725 | 0.1412 | 0.0448 | 0.0142 | 0.003 |  |  |
| Phenylalanine | *C-1-2* | 302 | 0.3419 | 0.3243 | 0.2454 | 0.0657 | 0.0194 | 0.0034 |  |  |  |
| Phenylalanine | *C-2-3-4-5-6-7-8-9* | 308 | 0.0729 | 0.1332 | 0.2184 | 0.2404 | 0.1817 | 0.0986 | 0.0389 | 0.0123 | 0.0037 |
| Glutamate | *C-2-3-4-5* | 404 | 0.1512 | 0.2658 | 0.2968 | 0.1992 | 0.087 |  |  |  |  |
| Alanine | *C-2-3* | 232 | 0.392 | 0.3904 | 0.1617 | 0.0438 | 0.0102 | 0.0014 | 0.0003 | 0.0002 |  |
| Aspartate | *C-2-3-4* | 390 | 0.216 | 0.3061 | 0.2735 | 0.1381 | 0.047 | 0.0139 | 0.003 | 0.0015 | 0.0009 |
| Aspartate | *C-1-2* | 302 | 0.3306 | 0.3251 | 0.2495 | 0.068 | 0.0213 | 0.0044 | 0.0013 |  |  |
| Valine | *C-2-3-4-5* | 260 | 0.2112 | 0.3472 | 0.2705 | 0.1222 | 0.0373 | 0.0092 | 0.0017 | 0.0005 | 0.0002 |

**References**

1. Antoniewicz MR, Kelleher JK, Stephanopoulos G. Determination of confidence intervals of metabolic fluxes estimated from stable isotope measurements. Metabolic Engineering. 2006;8(4):324-37. doi: <http://dx.doi.org/10.1016/j.ymben.2006.01.004>.
