# Supplementary material for: Metabolic flux analysis of heterotrophic growth in Chlamydomonas reinhardtii
Source: PLoS One. 2017 May 24;12(5):e0177292. doi: 10.1371/journal.pone.0177292 (PMC5443493; doi:10.1371/journal.pone.0177292)
Supplement: S7 Table — (DOCX) [file pone.0177292.s010.docx]

**S7 Table. Additional Isotope distribution of TMS derivatives of sugar fragments as described in MacLeod et al. [**[**1**](#_ENREF_1)**]**

|  | **Fragment** | **Derived from** | **Mass** | **M+0** | **M+1** | **M+2** | **M+3** | **M+4** | **M+5** | **M+6** |
| --- | --- | --- | --- | --- | --- | --- | --- | --- | --- | --- |
| Xylose | *C-4-5* | Cell wall | 205 | 0.422 | 0.3168 | 0.2069 | 0.0543 |  |  |  |
| Xylose | *C-1-2-3* | Cell wall | 262 | 0.2612 | 0.3171 | 0.252 | 0.1226 | 0.0471 |  |  |
| Glucose | *C-5-6* | Starch | 205 | 0.3986 | 0.3864 | 0.1616 | 0.0431 | 0.0103 |  |  |
| Glucose | *C-1-2-3* | Starch | 262 | 0.2214 | 0.3062 | 0.2306 | 0.1492 | 0.0706 | 0.022 |  |
| Glucose | *C-1-2-3-4* | Starch | 364 | 0.0937 | 0.245 | 0.302 | 0.2149 | 0.1004 | 0.0342 | 0.0098 |

**References**

1. MacLeod JK, Flanigan IL, Williams JF, Collins JG. Mass spectrometric studies of the path of carbon in photosynthesis: positional isotopic analysis of ^13^C-labelled C_4_ to C_7_ sugar phosphates. Journal of Mass Spectrometry. 2001;36(5):500-8. doi: 10.1002/jms.147.
