# Supplementary material for: Metabolic flux analysis of heterotrophic growth in Chlamydomonas reinhardtii
Source: PLoS One. 2017 May 24;12(5):e0177292. doi: 10.1371/journal.pone.0177292 (PMC5443493; doi:10.1371/journal.pone.0177292)
Supplement: S8 Table — OA and MAL are lumped, as are PEP and 3PG. (DOCX) [file pone.0177292.s011.docx]

**S8 Table. Coefficients for biomass formation equation used in ^13^C-MFA and FBA calculations given in moles per kilogram biomass.** OA and MAL are lumped, as are PEP and 3PG.

| **Metabolite** | **Stoich Coefficient for 1 kg** |
| --- | --- |
| G6P | 2.54 |
| αKG | 0.75 |
| OA | 0.62 |
| PYR | 1.31 |
| PEP | 0.28 |
| E4P | 0.08 |
| R5P | 0.12 |
| AcCoA | 6.85 |
| GAP | 0.42 |
