# Supplementary material for: Metabolic flux analysis of heterotrophic growth in Chlamydomonas reinhardtii
Source: PLoS One. 2017 May 24;12(5):e0177292. doi: 10.1371/journal.pone.0177292 (PMC5443493; doi:10.1371/journal.pone.0177292)
Supplement: S9 Table — Flux values in mol/10 mol of acetate. (DOCX) [file pone.0177292.s012.docx]

**S9 Table. Estimated normalized net fluxes, exchange coefficients and G parameters for Case B with standard deviation(s.d.), estimated from 95% intervals.** Flux values in mol/10 mol of acetate

| **Net Fluxes** | **Value** | **s.d** |
| --- | --- | --- |
| v33_77 | 6.48 | ±2.29 |
| v45_46 | 0.44 | ±0.41 |
| v1 | 0.00 | ±0.05 |
| v55 | 0.17 | ±0.06 |
| v30_29 | 2.10 | ±2.29 |
| v68 | 1.14 | ±0.98 |
| v51_52 | 0.21 | ±0.06 |
| v70 | 5.30 | ±2.26 |
| v6_7 | 0.21 | ±0.12 |
| v13_12 | 1.18 | 0.34 |
| v40_v41 | 1.38 | 1.64 |
| v38_39 | -0.65 | 0.37 |
| v80_81 | 12.99 | 3.31 |
| **Exchange Coefficients** |  |  |
| Vh18_71 | 0.055 | ±0.242 |
| vh33_77 | 0.334 | ±0.204 |
| vh14_76 | 0.800 | ±0.204 |
| vh31_32 | 0.776 | ±0.204 |
| vh35_75 | 0.083 | ±0.008 |
| vh80_81 | 0.126 | ±0.031 |
| vh2_3 | 0.104 | ±0.202 |
| vh10_11 | 0.044 | ±0.203 |
| vh38_39 | 0.000 | ±0.154 |
| vh51_52 | 0.002 | ±0.190 |
| vh29_30 | 0.565 | ±0.204 |
| vh12_13 | 0.800 | ±0.204 |
| vh42_43 | 0.000 | ±0.204 |
| vh4_5 | 0.000 | ±0.055 |
| vh6_7 | 0.800 | ±0.204 |
| vh36_37 | 0.605 | ±0.204 |
| vh40_41 | 0.000 | ±0.160 |
| vh49_50 | 0.000 | ±0.204 |
| vh53_54 | 0.036 | ±0.201 |
| vh47_48 | 0.770 | ±0.204 |
| vh8_9 | 0.516 | ±0.204 |
| vh45_46 | 0.765 | ±0.204 |
| vh27_28 | 0.800 | ±0.204 |
| **G parameter** |  |  |
| G_leu | 0.916 | ±0.007 |
| G_Ile | 0.983 | ±0.005 |
| G_Phe | 0.939 | ±0.010 |
| G_Glu | 0.872 | ±0.009 |
| G_Thr | 0.947 | ±0.000 |
| G_AcoA | 0.983 | ±0.000 |
| G_hexp (hexose plastid pool | 0.946 | ±0.000 |
| G_hexc ( hexose cytosol pool) | 0.881 | ±0.000 |
| G_penc (pentose cytosol pool) | 0.879 | ±0.000 |
| G_ala | 0.890 | ±0.000 |
| G_asp | 0.941 | ±0.009 |
| G_val | 0.892 | ±0.023 |
