# Supplementary material for: Metabolic flux analysis of heterotrophic growth in Chlamydomonas reinhardtii
Source: PLoS One. 2017 May 24;12(5):e0177292. doi: 10.1371/journal.pone.0177292 (PMC5443493; doi:10.1371/journal.pone.0177292)
Supplement: S10 Table — (DOCX) [file pone.0177292.s013.docx]

S10 Table. The calculated F-statistic and statistical significance from comparing the different network topologies.

| **Cases Compared** | **F-Statistic Calculated** | **N1** | **N2** | **p value** | **Comment** |
| --- | --- | --- | --- | --- | --- |
| A Vs B | 3.76 | 1 | 104 | 0.06 | B is better at 0.94 |
| B Vs C | 0.20 | 2 | 102 | 0.82 | C is not statistically better than B |
| C Vs D | 8.38 | 2 | 104 | 4.0 x 10^-4^ |  |
| D Vs E | 20.25 | 1 | 105 | 1.53 x 10^-5^ |  |
| A Vs E | Same Degrees of freedom , A has a better fit, F Stat cannot be computed | | | | |
| B VS D | Same Degrees of freedom , B has a better fit, F Stat cannot be computed | | | | |
